# Analysis of genome-wide association data by large-scale Bayesian logistic regression

**DOI:** 10.1186/1753-6561-3-s7-s16

**Published:** 2009-12-15

**Authors:** Yuanjia Wang, Nanshi Sha, Yixin Fang

**Affiliations:** 1 Department of Biostatistics, School of Public Health, Columbia University, 722 West 168th Street, New York, NY 10032, USA; 2 Department of Mathematics and Statistics, Georgia State University, 750 COE, 7th Floor, 30 Pryor Street, Atlanta, GA 30303, USA

## Abstract

Single-locus analysis is often used to analyze genome-wide association (GWA) data, but such analysis is subject to severe multiple comparisons adjustment. Multivariate logistic regression is proposed to fit a multi-locus model for case-control data. However, when the sample size is much smaller than the number of single-nucleotide polymorphisms (SNPs) or when correlation among SNPs is high, traditional multivariate logistic regression breaks down. To accommodate the scale of data from a GWA while controlling for collinearity and overfitting in a high dimensional predictor space, we propose a variable selection procedure using Bayesian logistic regression. We explored a connection between Bayesian regression with certain priors and *L*_1 _and *L*_2 _penalized logistic regression. After analyzing large number of SNPs simultaneously in a Bayesian regression, we selected important SNPs for further consideration. With much fewer SNPs of interest, problems of multiple comparisons and collinearity are less severe. We conducted simulation studies to examine probability of correctly selecting disease contributing SNPs and applied developed methods to analyze Genetic Analysis Workshop 16 North American Rheumatoid Arthritis Consortium data.

## Background

Single-locus analysis is a widely used approach to analyze genome-wide association (GWA) data, but it may not be adequate to capture complex pattern of disease etiology [1] and is subject to severe multiple comparisons adjustment, especially in a GWA, in which the typical number of comparisons made is hundreds of thousands. Methods to handle large number of single-nucleotide polymorphisms (SNPs) simultaneously are in demand. Logistic regression is a popular tool to assess association between a dichotomous trait and SNP genotypes. To analyze multiple SNPs simultaneously by logistic regression, one can include all SNPs of interest as predictors. A challenge of applying such approaches to GWA data is that the sample size is usually much smaller than the number of SNPs. Traditional multivariate logistic regression breaks down in this case. Another disadvantage of such an approach is that when the correlation between SNPs is high due to linkage disequilibrium (LD), the estimated coefficients are highly variable and the method performs poorly.

To accommodate large number of SNPs from a GWA while controlling for collinearity and overfitting in a high dimensional predictor space, we propose a variable selection procedure using Bayesian logistic regression. We explored a connection between certain priors and penalized logistic regression. After analysing large number of SNPs simultaneously in a Bayesian logistic regression, we selected important SNPs for further consideration. With much fewer selected SNPs of interest, problems of multiple comparisons and collinearity are less severe. We conducted simulation studies to examine the probability of correctly selecting disease contributing SNPs. Finally, we applied the methods to analyze Genetic Analysis Workshop (GAW) 16 Problem 1 chromosome 9 data.

## Methods

Logistic regression is commonly used to fit dichotomous dependent variables. The general form of logistic regression is:

Maximum likelihood is used to estimate parameters in the model. When the number of predictors exceeds the sample size, traditional logistic regression breaks down. In addition, when the predictors are high correlated, the maximum likehood estimate from Eq. (1) is of poor quality.

### Gaussian prior and *L*_2 _penalty

In a Bayesian logistic regression, the coefficients *β*_*j *_in Eq. (1) follows some prior distribution. There is a connection between the Gaussian prior  and the *L*_2 _penalized logistic regression. To be specific, if we assume *β*_j _is independent and follows a Gaussian distribution with mean 0 and variances *σ*_*j*_^2^, then finding the posterior mode of *β *is equivalent to maximizing the log likelihood of logistic regression with *L*_2 _penalty [2]. The prior variance *σ*_*j*_^2 ^represents the prior belief of whether *β*_*j *_will be near zero. A small value of *σ*_*j*_^2 ^indicates that *β*_*j *_is close to zero, and a large value indicates a less informative prior belief. Here we assume all *σ*_*j*_^2 ^have a common value *σ*^2^. *L*_2 _penalized logistic regression is proposed to deal with the problem of overfitting and collinearity for large number of predictors [3]. It minimizes the negative log-likelihood subject to a constraint on the *L*_2_-norm of the coefficients, that is, to minimize

where *l *is the log likelihood of the data. Choosing prior variances *σ*^2 ^is equivalent to choosing smoothing parameter *λ*. This is also the ridge regression.

### Laplace prior and *L*_1 _penalty

If we assume that *β*_*j *_is independent and follows a Laplace prior (*l*(*β*_*j*_|*τ*_*j*_) = *τ*_*j*_/2exp(-*τ*_*j*_|*β*_*j*_|)) in a Bayesian logistic regression, then finding the posterior mode of *β *is equivalent to minimizing the negative log likelihood of logistic regression with *L*_1 _penalty, which is

While *L*_2 _penalized regression shrinks coefficients towards zero, it does not favor them to be exactly zero. In contrast, *L*_1 _penalized regression provides sparse solutions when a large number of coefficients will be zero. Here we assume the prior parameter *τ*_j _to take the common value *τ*. This is also the LASSO regression.

### Selecting prior parameters

Choosing prior variance of the parameters in a Bayesian regression, or equivalently, the regularization parameter in a penalized regression, is important for variable selection. A small prior variance provides more shrinkage towards zero or favors more coefficients to be zero. A large prior variance reflects more uncertainty of the prior information. The prior variance was chosen by 10-fold cross validation. The sample was split randomly into 10 parts. The model was fit on 9 out of the 10 parts and the log likelihood function was computed using the remaining one part of the data. This procedure was done for each of the 10 parts and the average log likelihood was calculated. The prior variance was chosen as the one that maximizes the "cross-validated" average log likelihood.

### Simulations

We performed simulation studies to examine the effectiveness of Bayesian logistic regression as a variable selection procedure. We simulated 100 dichotomous predictors from a Bernoulli distribution. The probability of the predictor being one is generated from a uniform distribution, *U*(0.25, 0.45). Ten of the hundred predictors jointly determine a subject's disease status. The remaining 90 predictors are not used in simulating subjects' disease status. We simulated two settings of sample sizes (*n *= 150 and *n *= 250) and two settings of odds ratios. The odds ratios are simulated from a uniform distribution, *U*(1.5, 2), or *U*(2, 2.5).

We fit Bayesian logistic regression with Gaussian and Laplace priors using software BBRBMR [4]. BBRBMR can fit large-scale regressions with tens of thousands of predictors in a timely fashion. The algorithms used find posterior mode of a logistic likelihood efficiently [4]. We chose the prior variances by 10-fold cross validation. The logistic regression with Gaussian prior does not do variable selection directly. After performing the Bayesian analysis of all SNPs together, we selected SNPs for the second stage analysis by ranking their estimated regression coefficients from the first stage simultaneous SNP analysis. We simulated 30 sets of data under each of the four combinations of sample size and odds ratio. The effectiveness of proposed methods is evaluated by 1) the average number of disease-contributing predictors selected (out of the ten); and 2) how consistent each of the ten predictors is selected. The consistency is defined as the average percent times of each disease-contributing variable being selected across simulation data sets.

### NARAC data analysis

All analyses were performed on the GAW16 Problem 1 North American Rheumatoid Arthritis Consortium (NARAC) data. We analyzed 2705 SNPs on chromosome 9, ranging from 91,730,970 kb to 138,303,776 kb with minor allele frequency greater than 0.01 and no missing genotypes. This area covers the location where the most significant SNP (rs3761847) was reported by Plenge et al. [5]. We checked all 2705 SNPs for Hardy-Weinberg equilibrium (HWE) in the controls using PLINK [6] and did not find any SNP significantly violate HWE assumption after using the Bonferroni adjustment for multiple comparisons. The SNPs were coded in two ways: dominant and additive.

We divided the sample into a discovering sample (*N *= 1031) and a replication sample (*N *= 1031). First, we fit Bayesian logistic regression with a Gaussian prior using BBRBMR software on the training sample. We bootstrapped 100 times to provide standard error of the estimated coefficients. Second, we selected the top 300 SNPs according to two criteria: 1) the absolute value of the coefficients, and 2) the ratio of the coefficients to their bootstrapped standard errors (*z *scores). Selecting variables based on the absolute value of the coefficients instead of *z *scores may provide more reproducible results [7]. Especially for the SNPs with large signals and large variability, the *z *score may be low, but the coefficient may be large. We compare results using these two selection criteria. Third, we conducted chi-square tests on the 300 selected top-ranking SNPs using the independent testing sample. We analyzed data under both a dominant and additive model.

## Results

### Simulations

For the Gaussian prior with sample size 250 and high odds ratio (odds ratio ranging from 2 to 2.5), the average number of correctly identified SNPs in the top 20 SNPs selected by the magnitude of the regression coefficients is 8.3 (out of the 10 disease-associated SNPs). For the same prior and the sample size but with moderate odds ratio (odds ratio ranging from 1.5 to 2), the average number of correctly identified SNPs is 6.7. When decreasing the sample size to 150, in the high and moderate odds ratio model, the average number of correctly identified SNPs is 7.4 and 6.4, respectively. The consistencies (the average percent times of each disease-contributing variable being selected across simulation data sets) in the above four settings ranges from 0.73 to 0.97, 0.53 to 0.77, 0.6 to 0.87, and 0.57 to 0.73. For the Laplace prior, the average numbers of SNPs correctly identified in each of the four settings were: 6.7, 4.5, 4.2, and 4.0, respectively. The consistencies were lower than the Gaussian prior.

### NARAC data analysis

**Figure 1:**
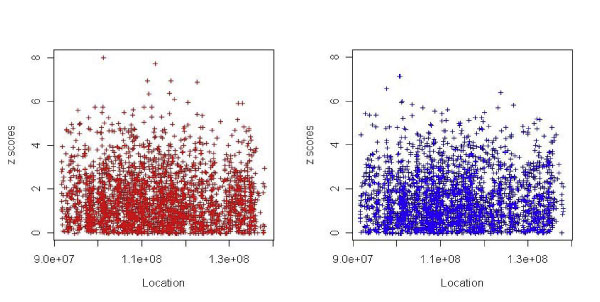
***p-*Values for 2705 SNPs in the Bayesian logistic regression (Gaussian prior): additive model (left panel) and dominant model (right panel)**.

For the Bayesian logistic regression with 2705 SNPs, the number of iteration in the Markov-Chain Monte Carlo calculation was 250 for the additive model and 187 for the dominance model. Convergence was reached with threshold 0.005. For the dominant model, the highest *z *score was 7.16 (rs7864653 at 100,860,678 kb). For the additive model, the highest *z *score was 8.02 (rs1407869 at 101,353,456 kb). Figure 1 displays the *z *scores for all 2705 SNPs. Table 1 shows numerical results of the highest ranked SNPs. Several top-ranked SNPs lie in the region where the most significant SNP was reported by Plenge et al. [5] (rs3761847 at 120,769,793 kb): for example, rs2900180 at 120,785,936 kb and rs1953216 at 120,720,054 kb.

**Table 1:** Bayesian logistic regression of 2705 SNPs on chromosome 9

	Additive model			Dominant model		
		
Rank	SNP	Position	abs (*z-*score)	SNP	Position	abs (*z*-score)
1	rs1407869	101353456	8.02	rs7864653	100860678	7.16
2	rs4437724	113188649	7.76	rs10989329	100794635	7.16
3	rs10120479	111426956	6.97	rs4237190	97922972	6.58
4	rs9697192	116879138	6.97	rs6478644	123942505	6.42
5	rs3824535	122763410	6.90	rs1407869	101353456	6.03
6	rs10491578	116463442	6.39	rs2229594	101204219	5.97
7	rs10121681	111718477	6.37	rs10820559	103716588	5.87
8	rs694428	117692812	6.13	rs1536705	126851425	5.86
9	rs2900180	120785936	5.96	rs2564362	123365200	5.74
10	rs11243755	132287257	5.96	rs10978456	106155366	5.73

**Figure 2:**
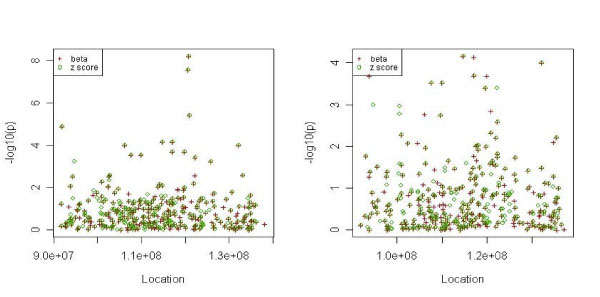
***p-*Values of the top 300 SNPs selected by *β *or *z *scores (single-SNP analysis): additive model (left panel) and dominant model (right panel)**.

We selected the top 300 SNPs and performed single-SNP analysis using the independent testing set of 1031 subjects. LD plot revealed that the selected SNPs had lower intermarker LD than the total marker map (not shown). Table 2 summarizes the top 10 SNPs with the lowest *p-*values in each model. The top three SNPs in the additive model were in the region reported by Plenge et al. [5]. Instead of selecting by *z *scores, we also selected the top 300 SNPs by the absolute value of the regression coefficients. For the additive model, selecting by absolute value of *β *or by *z *score provided the same ranking for the top 14 SNPs. For the dominant model, there were 10 overlapping SNPs for the two selection criteria among the top 15 SNPs. Figure 2 depicts the *p-*values of the SNPs selected by the two criteria: circles for the *z *score method and crosses for *β-*based method. The two criteria selected similar sets of SNPs.

**Table 2:** Single-SNP analysis of the top 300 selected SNPs

	Additive model			Dominant model		
		
Rank	SNP	Position	*p-*Value	SNP	Position	*p-*Value
1	rs2900180	120785936	6.24 × 10^-9^	rs2900180	120785936	6.24 × 10^-9^
2	rs1953126	120720054	2.76 × 10^-8^	rs11787779	114820894	6.89 × 10^-5^
3	rs942152	121031239	3.94 × 10^-6^	rs17148869	132180015	1.00 × 10^-4^
4	rs7858974	91959665	1.26 × 10^-5^	rs7862566	117133575	2.00 × 10^-4^
5	rs11787779	114820894	6.89 × 10^-5^	rs4978629	107708375	3.00 × 10^-4^
6	rs6478300	117115323	7.12 × 10^-5^	rs4978890	110046695	3.00 × 10^-4^
7	rs989980	106309592	1.00 × 10^-4^	rs1333914	119662788	4.00 × 10^-4^
8	rs17148869	132180015	1.00 × 10^-4^	rs1332408	122271713	4.00 × 10^-4^
9	rs7862566	117133575	2.00 × 10^-4^	rs2095069	94782055	0.001
10	rs945246	119953710	2.00 × 10^-4^	rs4743420	100567644	0.0011

## Discussion

We propose a Bayesian logistic regression procedure to select important SNPs based on the *z *scores or the regression coefficient estimates for further analysis. From the simulation studies, when using a Gaussian prior, the percentage of causal SNPs correctly selected ranges from 64% to 83% among the top 20% SNPs. For the Laplace prior, the percentage of correctly identified causal SNPs ranges from 40% to 67%. The Gaussian prior outperforms Laplace prior, which could be attributable to a less stringent feature selection criterion employed for the Gaussian prior.

Among the top 300 SNPs selected by the *z *scores for the dominance model, three are significant after adjusting for multiple comparisons (see Table 2). For the additive model, five additional SNPs are significant after multiple comparisons adjustment. These SNPs lie in a region from 91,959,665 kb to 132,180,015 kb on chromosome 9 (LD plots not included due to space limitations). Three of the eight SNPs are in the region reported in Plenge et al. [5] (rs1953126, rs2900180, and rs942152), and two of them are in LD (rs1953126 and rs2900180). One of these SNPs, rs1953126, was reported in a study of 475 Caucasian patients [8] to be significantly associated with rheumatoid arthritis (odds ratio 1.28, CI 1.16-1.40, trend *p-*value = 1.45 × 10^-6^). The other five SNPs are not in the candidate region and are not in LD with SNPs in the region. The significance of other SNPs deserves further investigation in an independent sample.

An alternative one-step approach would be reporting permutation *p*-values of Bayesian logistic regression with all SNPs on the whole sample. However, it is well known that increasing number of predictors, and therefore the number of parameters, in a multivariate analysis may reduce power. The two-step approach provides a balance between the need to reduce multiple comparisons and the loss of power due to increasing number of parameters.

We only analyzed SNPs with no missing data due to the incapability of handling missing covariates data of the BBRBMR software. One solution is to first impute the missing genotypes and then run the Bayesian regression on the imputed data. An alternative is to handle missing data directly in a Bayesian analysis by data augmentation.

Here the priors are assumed to be independent and their variances are assumed to be the same. We choose prior variance by cross-validation. An alternative strategy would be specifying a hyper-prior distribution (such as non-informative prior). To incorporate prior knowledge such as physical distance between the SNPs, one can specify prior distribution to have distance-based correlation. How to specify such a correlation for a large scale regression is worth further attention.

## Conclusion

Large scale Bayesian logistic regression is useful to analyze genome wide case-control data with large number of SNPs. Coefficient estimates or *z *scores from such regression can be used to select important SNPs for further genetic analysis. Such procedure reduces number of tests performed and alleviates problem of multiple comparisons.

## List of abbreviations used

GAW: Genetic Analysis Workshop; GWA: Genome-wide association; HWE: Hardy-Weinberg equilibrium; LD: Linkage disequilibrium; NARAC: North American Rheumatoid Arthritis Consortium; SNP: Single-nucleotide polymorphism

## Competing interests

The authors declare that they have no competing interests.

## Authors' contributions

YW designed the study, performed the statistical analysis, and drafted the manuscript. NS performed the statistical analysis. YF participated in the study design and helped to draft the mansucript. All authors read and approved the final manuscript.

## References

[B1] Hoh J Ott J Mathematical multi-locus approaches to localizing complex human trait genes Nat Rev Genet 2003 4 701 709 10.1038/nrg1155 12951571

[B2] Santner T Duffy D The Statistical Analysis of Discrete Data 1989 New York, Springer

[B3] Park MY Hastie T Penalized logistic regression for detecting gene interactions Biostat 2008 9 30 50 10.1093/biostatistics/kxm010 17429103

[B4] Genkin A Lewis D Madigan D Large-scale Bayesian logistic regression for text categorization Technometrics 2007 49 291 304 10.1198/004017007000000245

[B5] Plenge RM Seielstad M Padyukov L Lee AT Remmers EF Ding B Liew A Khalili H Chandrasekaran A Davies LR Li W Tan AK Bonnard C Ong RT Thalamuthu A Pettersson S Liu C Tian C Chen WV Carulli JP Beckman EM Altshuler D Alfredsson L Criswell LA Amos CI Seldin MF Kastner DL Klareskog L Gregersen PK TRAF1-C5 as a risk locus for rheumatoid arthritis--a genomewide study N Engl J Med 2007 357 1199 1209 17804836 10.1056/NEJMoa073491 PMC2636867

[B6] Purcell S Neale B Todd-Brown K Thomas L Ferreira MAR Bender D Maller J Sklar P de Bakker PIW Daly MJ Sham PC PLINK: a tool set for whole-genome association and population-based linkage analyses Am J Hum Genet 2007 81 559 575 17701901 10.1086/519795 PMC1950838

[B7] Guo L Lobenhofer EK Wang C Shippy R Harris SC Zhang L Mei N Chen T Herman D Goodsaid FM Hurban P Phillips KL Xu J Deng X Sun YA Tong W Dragan YP Shi L Rat toxicogenomic study reveals analytical consistency across microarray platforms Nat Biotechnol 2006 24 1162 1169 10.1038/nbt1238 17061323

[B8] Chang M Rowland CM Garcia VE Schrodi SJ Catanese JJ Helm-van Mil AH van der Ardlie KG Amos CI Criswell LA Kastner DL Gregersen PK Kurreeman FA Toes RE Huizinga TW Seldin MF Begovich AB A large-scale rheumatoid arthritis genetic study identifies association at chromosome 9q33.2 PLoS Genet 2008 4 e1000107 18648537 10.1371/journal.pgen.1000107 PMC2481282

